# Cancer mortality in childhood and adolescence: analysis of trends and spatial distribution in the 133 intermediate Brazilian regions grouped by macroregions

**DOI:** 10.1590/1980-549720240003

**Published:** 2024-01-26

**Authors:** Kamila Tessarolo Velame, José Leopoldo Ferreira Antunes

**Affiliations:** I Universidade de São Paulo, School of Public Health – São Paulo (SP), Brazil.

**Keywords:** Epidemiology, Cancer, Mortality, Time series, Epidemiologia, Câncer, Mortalidade, Séries temporais

## Abstract

**Objective:**

To assess the magnitude, trend, and spatial patterns of childhood and adolescent cancer mortality between 1996 and 2017 in 133 Brazilian intermediate regions by using socioeconomic and healthcare services indicators.

**Methods:**

This is an ecological study for analyzing the trend of mortality from cancer in childhood and adolescence through time series. Data on deaths were extracted from the Brazilian Mortality Information System. Data on population were extracted from the 1991, 2000, and 2010 demographic censuses of the Brazilian Institute of Geography and Statistics, with interpolation for intercensal years. Time series were delineated for mortality by type of cancer in each intermediate region. Such regions were grouped by macroregions to present the results. The calculation and interpretation of mortality trends use the Prais-Winsten autoregression procedure.

**Results:**

Mortality rates for all neoplasms were higher in the Northern region (7.79 deaths per 100 thousand population), while for leukemias, they were higher in the Southern region (1.61 deaths per 100 thousand population). In both regions, mortality was higher in boys and in the 0-4 age group. The trend was decreasing (annual percent change [APC] – -2.11 [95%CI: -3.14; - 1.30]) for all neoplasms in the Brazilian regions and stationary (APC – -0.43 [95%CI: -1.61; 2.12]) for leukemias in the analyzed period.

**Conclusion:**

The mortality rate for all neoplasms showed higher values in regions with smaller numbers of ICU beds in the public healthcare system.

## RESUMO

**Objetivo:**

Avaliar magnitude, tendência e padrões espaciais da mortalidade por câncer na infância e adolescência, entre 1996 e 2017, nas 133 regiões intermediárias brasileiras, utilizando indicadores socioeconômicos e de serviços de saúde.

**Métodos:**

É um estudo ecológico que analisa a tendência da mortalidade por câncer na infância e adolescência por meio de séries temporais. Os dados sobre óbitos foram extraídos do Sistema de Informações sobre Mortalidade. Os dados referentes à população foram extraídos dos censos demográficos de 1991, 2000 e 2010 do Instituto Brasileiro de Geografia e Estatística, com interpolação para os anos intercensitários. Foram delineadas séries temporais para a mortalidade por tipo de câncer em cada região intermediária. Tais regiões foram agrupadas por macrorregiões para a apresentação dos resultados. O cálculo e interpretação das tendências de mortalidade utilizaram o procedimento de autorregressão de Prais-Winsten.

**Resultados:**

As taxas de mortalidade para todas as neoplasias foram maiores na Região Norte (7,79 óbitos por 100 mil habitantes), enquanto para as leucemias foram maiores na Região Sul (1,61 óbitos por 100 mil habitantes). Em ambas a mortalidade foi maior no sexo masculino e na faixa etária de zero a quatro anos. A tendência mostrou-se decrescente (annual percent change — APC -2,11 [intervalo de confiança — IC95% -3,14; -1,30]) para todas as neoplasias nas regiões brasileiras e estacionária (APC -0.43 [IC95%: -1.61; 2.12]) para as leucemias, no período analisado.

**Conclusão:**

A taxa de mortalidade, para todas as neoplasias, apresentou valores mais elevados nas regiões que dispõem de menores números de leito de Unidade de Terapia Intensiva do Sistema Único de Saúde.

## INTRODUCTION

Cancer in childhood and adolescence, a serious public health issue, accounts for 2 to 3% of all cancers in Brazil^1^. In low- and middle-income countries, cancers in childhood and adolescence are often fatal due to the lack of early diagnosis and treatment. This occurs due to the lack of population screening programs or lifestyle-related risk reduction strategies that are more effective in obtaining favorable results. Childhood cancer, therefore, is often fatal when appropriate diagnosis and treatment are not carried out. Childhood cancer usually progresses quickly. Therefore, improving childhood cancer outcomes requires well-functioning health systems^2^.

However, for the underreporting and the quality of data from the Brazilian Mortality Information System (*Sistema de Informações sobre Mortalidade* – SIM) to improve not only in some Brazilian regions, but in all of them, there must be adequate planning on the part of the responsible political institutions, in such a way that there is an adequate allocation of resources to the cause in question and, consequently, better functioning of the Brazilian health system. It is essential that models and estimates are developed where data are scarce or perhaps nonexistent^2^.

Such variations are the subject of discussion in the literature, which correlates data with differences in socioeconomic indicators between populations in developed and developingcountries^3^. Regarding Brazil, for example, data may differ across regions due to improved information made available to the scientific community as well as variations in the organization of the health system. However, the literary consensus is that these regional differences cannot exist, as they affect health indicators and lead to a disproportionate loss of human capital (children and adolescents)^4^.

Among the types of cancer, leukemia is the most common, especially in children under five years of age, accounting for between 25 and 35% of all types^1^. In the study conducted by Ren et al.^5^, leukemia was also responsible for the highest burden of cancer in children under five years of age in 2019, followed by brain and central nervous system cancer. Leukemia was also the most frequently diagnosed incident cancer in 1990, although this number decreased considerably between 1990 and 2019 in developed countries. The number of deaths and burden from childhood cancer, respectively, decreased by -47.8% (-60.7 to -26.4) in 1990; and by -47.7% (-60.7 to -26.2 ) in 2019. In 2019, regions with a medium sociodemographic index had the highest incidence and prevalence, whereas regions with a low socioeconomic index had the highest mortality and burden.

The global trend, therefore, is an increase in the incidence of childhood cancer, which can also be explained by improvements in information systems and a reduction in mortality rates thanks to early diagnosis and treatment in several regions^2^. Statistics on childhood neoplasms are little analyzed in Brazil, even with the availability of databases, as in the case of the SIM. Although research on temporal trends is extensive in adults, few studies have been carried out on children and adolescents in the recent decades^6^.

The importance of evaluating geographic variations in cancer mortality rates in childhood and adolescence is justified by the territorial extension of Brazil and its great diversity in terms of access to cancer diagnosis and treatment. Overall, these resources are insufficient and are centralized in the capitals and in the most economically developed states. The mismatch between the place of residence and the location of the health service warns of access problems and impacts on information systems^7^.

Hence, the objective of this study was to assess the magnitude, trend, and spatial patterns of childhood and adolescent cancer mortality between 1996 and 2017, in 133 Brazilian intermediate regions by using socioeconomic and healthcare services indicators and by grouping these regions into macroregions.

## METHODS

This is an ecological study that analyzes the trend in cancer mortality in childhood and adolescence through time series and jointly observes the spatial distribution of this mortality between 1996 and 2017, in the age group between zero and 19 years. In this study, time series trends were used, which allowed the comparison of cancer mortality rates in childhood and adolescence over time in geographically defined population groups.

Deaths from cancer in childhood and adolescence in intermediate Brazilian regions between 1996 and 2017 were analyzed. Data were extracted from the SIM. Deaths classified as malignant neoplasms were selected according to the Tenth Revision of the International Classification of Diseases (ICD-10), which comprises chapter II, group C00 to C99, including D46. Data on deaths were extracted using the file transfer system made available by the Informatics System of the Brazilian Unified Health System (DATASUS).

Data on the population were extracted from the 1991, 2000, and 2010 demographic censuses of the Brazilian Institute of Geography and Statistics (IBGE). For the intercensal years, from 1996 to 2017, population estimates were used according to sex and age group. Such data are available on the IBGE website. The intermediate regions were also defined according to the IBGE classification and, for the presentation of results, the intermediate regions were grouped by macroregions. The Crude Death Rate (CDR) was calculated annually by age group and by anatomical location (type of cancer), dividing the number of deaths due to childhood cancer by the population and multiplying this quotient by 100 thousand. To make mortality coefficients capable of comparison, standardized coefficients were calculated by sex and age group, using the direct method proposed by Laurenti et al.^8^

Socioeconomic information was based on data from the 2000 Demographic Census^9^. The analysis of socioeconomic data used the Municipal Human Development Index (HDI-M) for municipalities in Brazil. The HDI for the intermediate regions was estimated through the weighted average of the HDI-M of the municipalities in each intermediate region, using the population size of these municipalities as a weighting factor. Data on the provision of healthcare services were reported by information systems managed by the Brazilian Ministry of Health. Data from the National Registry of Health Establishments (*Cadastro Nacional de Estabelecimentos de Saúde* – CNES) and the Public Health Budget Information System (Sistema de Informações sobre Orçamentos Públicos em Saúde – SIOPS) were used in this study.

Time series were delineated for mortality by type of cancer in each intermediate region. The calculation and interpretation of mortality trends used the Prais-Winsten^10^ procedure for generalized linear regression, which allows the correction of first-order autocorrelation. For the trend analysis, methodological recommendations from Antunes and Cardoso^10^ were followed. The Prais-Winsten regression model was used; the dependent variable was the logarithm of cancer mortality rates in childhood and adolescence for all types of cancer and leukemias, and the independent variable was constituted by the years of the historical series (1996 to 2017).

The Annual Percent Change (APC) of rates standardized by age and sex was also calculated, as suggested by the study conducted by Antunes and Waldman^11^. In the modeling process, transformations of standardized rates into a base-10 logarithmic function were included. The Durbin-Watson test was used, which enabled the authors to verify the existence of first-order autocorrelation of the time series (as proposed by the Prais-Winsten regression), composed of annual rates, as well as to attest to the compatibility of the correlation carried out with the hypothesis of regression residuals with random distribution. Subsequently, the annual growth or decline rates (APC) were then calculated according to sex, age groups, and types of cancer (total and leukemia). This procedure enabled the authors to classify the temporal trend of cancer mortality in childhood and adolescence between zero and 19 years of age as decreasing, increasing, or stationary.

To construct the mortality time series, deaths initially classified as due to “ill-defined” or “unspecified” causes were redistributed according to the method proposed by the Global Burden of Disease (GBD) study^12^, which allows to estimate the proportion of these deaths that must be reclassified as attributable to cancer in each year and region, for each age group.

For database management and statistical analysis, the Stata 14.2 2017 software was used.

## RESULTS

Between 1996 and 2017, there were 62,635 deaths of children and adolescents whose underlying cause was identified as neoplasm. Of this total, 21,039 deaths were due to leukemia. After redistributing the deaths due to ill-defined causes, also known as garbage codes, the estimated total was 111,568 deaths for all neoplasms. With regard to leukemias, the estimate was 22,571.

**Figure 1.**
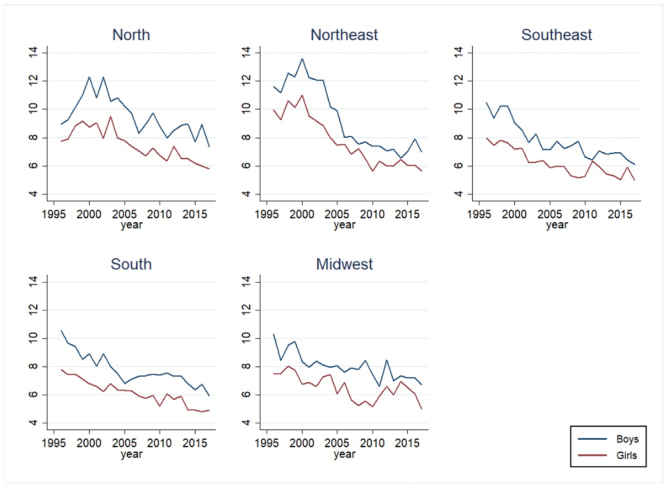
Mortality from all neoplasms in children and adolescents (per 100 thousand individuals): historical series of rates, by sex, in each macroregion. Standardized rates by sex and age group. Brazil, 1996–2017.

The evaluation of the historical series of mortality from all neoplasms in children and adolescents by sex shows a mortality rate for boys higher than that for girls in all Brazilian regions, with a decreasing trend in most regions. As shown in Figure 1, the Northeast region showed a significant decline throughout the historical series, despite its higher mortality rate at the beginning of the series (APC) -3.16 [95%CI -4.58; 0.37]).

The historical series of mortality from neoplasms in children and adolescents by age group demonstrates that from zero to four years of age, mortality rates are higher in all Brazilian regions. The ages of five to nine and ten to 14 years overlap throughout the series for all regions. From 15 to 19 years of age, these rates are lower than from zero to four years, although higher than those for the other two age groups.

Regarding the APC of mortality from all neoplasms, in Table 1 we show a decline in the number of deaths throughout the historical series in all regions . In other words, altogether, Brazil benefits from this decline (APC -2.11 [95%CI -3.14; -1.30]). More significant declines were observed in the Northeast (APC -3.16 [95%CI -4.58; 0.37]) and Southeast (APC -2.11 [95%CI -2.68; -1.59]) regions, and we observed the smallest decline in the Midwest region (APC -0.95 [95%CI -2.24; -0.63]).

**Table 1.** Annual Percent Change in mortality from all neoplasms in children and adolescents: median and interquartile range of the Intermediate Geographic Regions of Brazil, grouped by macroregion, and distribution of trends. Brazil, 1996–2017.

	APC: Median (IRQ)	Increasing n (%)	Decreasing n (%)	Stationary n (%)
North (n=22)	-1.75 (-2.77; -0.14)	2 (9.1)	12 (54.5)	8 (36.4)
Northeast (n=42)	-3.16 (-4.58; 0.37)	1 (2.4)	28 (66.7)	13 (30.9)
Southeast (n=33)	-2.11 (-2.68; -1.59)	0 (0.0)	27 (81.9)	6 (18.1)
South (n=21)	-1.82 (-2.49; -1.48)	0 (0.0)	14 (66.7)	7 (33.3)
Midwest (n=15)	-0.95 (-2.24; -0.63)	1 (6.6)	7 (46.7)	7 (46.7)
Brazil (n=133)	-2.11 (-3.14; -1.30)	4 (3.0)	88 (66.2)	41 (30.8)

APC: Annual Percent Change; IQR: Interquartile range.

Some variables were analyzed, such as HDI-M, coverage of the Family Health Strategy, health expenses, government health expenses on outpatients and hospital patients, total number of general beds, number of general beds maintained by the SUS, total number of beds in Intensive Care Units (ICU), and number of ICU beds maintained by the SUS. The Pearson’s correlation (R) was applied in this analysis to verify the presence of a linear association and the strength between the variables and the mortality rate. Only one variable was associated, the number of SUS ICU beds (R: -0.23; p=0.007), as shown in Table 2.

**Table 2.** Median Annual Percent Change in mortality from all neoplasms in children and adolescents, by quartile of variables, and Pearson’s correlation coefficient (R) between the Annual Percent Change and the variables. Brazil, 1996–2017.

Variable	1st quartile	2nd quartile	3rd quartile	4th quartile	R	p-value
Human Development Index	-2.32	-2.77	-2.06	-1.85	-0.12	0.165
Coverage by the Family Health Strategy	-1.88	-2.41	-2.11	-2.41	0.10	0.274
Government health expenses	-2.43	-2.41	-2.11	-1.82	0.01	0.899
Expenses on outpatients	-2.40	-1.85	-2.19	-1.89	-0.09	0.311
Expenses on hospital patients	-2.07	-2.72	-1.89	-1.73	0.01	0.871
Hospital beds	-2.36	-2.41	-1.58	-2.06	-0.04	0.621
ICU beds	-2.20	-2.48	-2.20	-1.91	-0.16	0.060
SUS hospital beds	-2.01	-2.24	-1.87	-2.13	0.07	0.430
SUS ICU beds	-1.82	-2.39	-1.88	-2.11	-0.23*	0.007*

ICU: intensive care unit; SUS: Brazilian Unified Health System. *Presence of linear association and strength between variables and mortality rate.

Regarding leukemias, there was a greater mortality from leukemia in boys than in girls in all Brazilian regions over the years. Furthermore, in the North region, mortality increases for every 100 thousand inhabitants in the final years, with the Southeast region remaining stationary. Children aged zero to four years had higher mortality rates in all regions of Brazil. In the South, the age group of 15 to 19 years exceeds that of zero to four years at a given moment; however, in the final years, it returns to the position below the age group of zero to four years of age.

We verified a stationary trend for Brazil as a whole. In the North region, of the 22 intermediate regions, 17 remained stationary throughout the series, and five were increasing. The Northeast region presented APC 0.27 (95%CI -1.08; 3.68) and, of the 42 intermediate regions, 28 remained stationary, 12 increased, and two decreased. The Southeast region had 30 intermediate regions with a stationary trend and three decreasing. In the South of the country, there were 17 intermediate regions with a stationary trend and four with a decreasing trend. Of the 15 intermediate regions in the Midwest, 12 remained stationary, two increased, and one decreased, as shown in Table 3.

**Table 3.** Annual Percent Change in mortality from all neoplasms in children and adolescents: median and interquartile range of the Intermediate Geographic Regions of Brazil, grouped by macroregion, and distribution of trends. Brazil, 1996–2017.

	APC: Median (IRQ)	Increasing: n (%)	Decreasing: n (%)	Stationary: n (%)
North (n=22)	1.55 (-1.46; 5.01)	5 (22.7)	0 (0.0)	17 (77.3)
Northeast (n=42)	0.27 (-1.08; 3.68)	12 (28.6)	2 (4.7)	28 (66.7)
Southeast (n=33)	-0.68 (-1.46; 1.11)	0 (0.0)	3 (9.0)	30 (91.0)
South (n=21)	-1.00 (-3.10; -0.41)	0 (0.0)	4 (19.0)	17 (81.0)
Midwest (n=15)	-0.27 (-2.88; 2.76)	2 (13.3)	1 (6.7)	12 (80.0)
Brazil (n=133)	-0.43 (-1.61; 2.12)	19 (14.3)	10 (7.5)	104 (78.2)

APC: Annual Percent Change; IQR: Interquartile range.

For leukemias, the association test, the Pearson’s correlation coefficient, was also used to verify the variables that somehow influence the mortality rate from leukemias in children and adolescents. Of the nine variables analyzed, five showed statistical significance, with a value of p<0.05, namely: HDI-M (R: -0.38; p<0.01), government health expenses on outpatients (R: -0.27; p<0.01), number of beds (R: -0.18; p=0.040), ICU beds (R: -0.21; p=0.015), and SUS ICU beds (R: -0.24; p=0.001), as shown in Table 4.

**Table 4.** Median Annual Percent Change in mortality from leukemia in children and adolescents, by quartile of variables, and Pearson’s correlation coefficient (R) between the Annual Percent Change and the variables. Brazil, 1996–2017.

Variable	1st quartile	2nd quartile	3rd quartile	4th quartile	R	p-value
Human Development Index	2.07	0.23	-0.83	-0.83	-0.38*	<0.01*
Coverage by the Family Health Strategy	-0.58	-0.71	0.88	0.17	0.16	0.06
Government health expenses	0.77	0.13	-0.50	-0.65	-0.13	0.141
Expenses on outpatients	2.34	-0.43	-0.71	-0.68	-0.27*	<0.01*
Expenses on hospital patients	0.55	-0.37	-0.40	-0.83	-0.07	0.439
Hospital beds	1.24	-0.65	0.15	-1.00	-0.18*	0.040*
ICU beds	1.36	-0.50	-0.42	-0.73	-0.21*	0.015*
SUS hospital beds	0.59	-0.68	0.23	-0.83	-0.01	0.940
SUS ICU beds	1.00	-0.48	-0.40	-0.83	-0.24*	0.001*

ICU: intensive care unit; SUS: Brazilian Unified Health System. *Of the nine variables analyzed, five showed statistical significance, with a value of p<0.05. HDI-M had an association of (R: -0.38; p<0.01).

## DISCUSSION

With regard to cancer mortality in children and adolescents, taking into account all neoplasms, we observed a change in the mortality rate throughout the studied period. The geographic patterns of cancer mortality in children and adolescents were also different, with a predominance in the North and Northeast regions. Not only was there a change in the mortality rate, but the APC was decreasing.

In Latin America, Brazil is the sixth country with the highest mortality rate from childhood cancer, only behind Cuba (4.93 deaths per 100 thousand inhabitants), Mexico (4.92), Colombia (4.87) , Panama (4.43), and Costa Rica (4.30)^13^. In this study, the cancer mortality rate in children and adolescents in Brazil was 7.33 deaths per 100 thousand inhabitants, 8.07 deaths for boys and 6.49 deaths for girls per 100 thousand inhabitants. The study by Malvezzi et al.^13^, previously mentioned, had recorded, for Brazil, a mortality rate of 4.04 deaths per 100 thousand inhabitants — 4.33 and 3.74, respectively, for boys and girls. The data differ from those reported here and, as possible factors to explain this difference, we mention the different reference periods of each study and the redistribution, in our study, of deaths classified as due to ill-defined and unspecified causes.

In Brazil, GBD 2017 Childhood Cancer Collaborators^2^ studied trends in childhood cancer mortality between 1979 and 2008, finding increasing trends in mortality rates for the North and Northeast regions. In this study, mortality rates in the North and Northeast regions were higher than those in other Brazilian regions, but the trends in these regions were decreasing. The GBD 2017 Childhood Cancer Collaborators^2^ also report that this situation is reflected in the unequal distribution of resources allocated or made available to health.

There is a disparity between areas with better urban structures (South and Southeast regions), with a well-equipped health team, and territories with scarce health resources such as the North and Northeast of the country.

It was also observed that residents of some more peripheral and northeastern locations, such as Maranhão, southern Piauí and western Bahia, have less access to chemotherapy and radiotherapy, compared to other Brazilian regions. Oncological surgeries and cancer hospitalizations showed similar patterns. For example, residents of the North and Northeast regions have less access to hospitalization and cancer surgery, which constitutes inequality in access. Places with fewer beds, especially ICU beds, imply a higher cancer mortality rate in children and adolescents.

The study by Curado et al.^14^ on leukemia mortality trends among children, adolescents, and young adults (zero to 24 years old) in Latin America detected, between 2000 and 2004, in Brazil, a mortality rate for females of 1.12 deaths per 100 thousand inhabitants and, for males, 1.77 deaths in the same proportion. Leukemia also shows a higher mortality rate for males, as previously demonstrated in other studies and in the results data obtained in this study.

Regarding the trend, mortality rates from leukemia in children and adolescents decreased (APC: -4.0) in Europe from 1990 to 2017^15^. The favorable trends are due to the increased availability of diagnosis and treatment, as well as the creation of centers specialized in pediatric oncology on the continent, leading to advances, with approximately 80% of patients having a survival of five years after diagnosis. Malvezzi et al.^13^ also observed a decreasing trend in mortality in children and adolescents (zero to 19 years old) due to leukemia between 1990 and 2017, with the exception of boys in Brazil and Colombia, where trends were stationary.

Bigoni et al.^16^, when seeking to describe mortality from the main types of cancer in adults in the 133 intermediate Brazilian regions, verified increasing trends for the North and Northeast regions, while increasing or stationary trends were observed in the South, Southeast and Midwest regions. One of the causes for these trends was the HDI. In regions with a higher HDI, greater government expenses on health and a greater number of hospital beds, mortality was lower. Furthermore, cancer mortality results from the lack of access to health care in neighboring and surrounding municipalities. Such findings reinforce the data from the results obtained in this study, in which the HDI-M, government health expenses on outpatients, the number of beds, the number of ICU beds, and the number of SUS ICU beds showed statistical significance, that is, they somehow contribute to leukemia mortality in children and adolescents.

Ribeiro et al.^17^ associate the mortality rate with social inequalities, once again confirming the results obtained in our study. Although the trend in mortality from childhood leukemia decreased in the surveyed years, the authors observed great heterogeneity between Brazilian states, closely related to socioeconomic development. Mortality had a sharper decrease in more developed states with better health care. Such findings point to the need to improve interventions, considering the widespread manifestation of social exclusion in Brazilian regions.

When evaluating the historical series, we observed that mortality in boys was higher than that of girls in all Brazilian regions, with a decreasing trend in most regions. The Northeast region showed a significant decline throughout the series, despite the mortality rate being higher at the beginning of the series. Furthermore, the mortality rate was higher in regions that have fewer SUS ICU beds and vice versa. Of the 133 intermediate regions, 88 showed a decreasing trend, 41 showed a stationary trend, and four showed an increasing trend.

As for leukemia, when considering the trends, we verified that the age group from zero to four years old had higher mortality rates than the others. There was stationarity for Brazil as a whole. Of the nine variables analyzed in the association test, five showed statistical significance, namely: IDH-M, government health expenses on outpatients, number of beds, number of ICU beds, and number of SUS ICU beds.

Regarding mortality from all neoplasms, we noticed a gap in secondary sources, with few studies, which demands the expansion of investigations in this line in order to contribute to the real understanding of the current context as well as providing subsidies for scholars of this area to seek the necessary changes to reduce these social and regional inequalities.

Other limitations of this study include the use of a single APC value to verify the trend throughout the entire analysis period. From 1996 to 2017, 22 points were included in the time series used to calculate the trend. In this sense, there may be discrepant trends if the analysis were segmented into different sub-periods. Thus, stationary trends in the results may have recently started to decrease, which a single APC value would not be able to detect. Moreover, increasing trends in childhood cancer mortality in less developed regions may have been influenced by an increase in data quality in the SIM over the years.
